# Host mRNA decay proteins influence HIV-1 replication and viral gene expression in primary monocyte-derived macrophages

**DOI:** 10.1186/s12977-019-0465-2

**Published:** 2019-02-07

**Authors:** Shringar Rao, Raquel Amorim, Meijuan Niu, Yann Breton, Michel J. Tremblay, Andrew J. Mouland

**Affiliations:** 1 HIV-1 RNA Trafficking Laboratory, Lady Davis Institute at the Jewish General Hospital, Montréal, Québec Canada; 2 Department of Microbiology and Immunology, McGill University, Montréal, Québec Canada; 3 Department of Medicine, McGill University, Montréal, Québec Canada; 4 Axe des Maladies Infectieuses et Immunitaires, Centre de Recherche du CHU de Québec-Université Laval, Québec, Québec Canada; 5 Département de Microbiologie-Infectiologie et Immunologie, Faculté de Médecine, Université Laval, Québec, Québec Canada

**Keywords:** HIV-1, Macrophages, UPF1, UPF2, SMG6, Staufen1

## Abstract

**Background:**

Mammalian cells harbour RNA quality control and degradative machineries such as nonsense-mediated mRNA decay that target cellular mRNAs for clearance from the cell to avoid aberrant gene expression. The role of the host mRNA decay pathways in macrophages in the context of human immunodeficiency virus type 1 (HIV-1) infection is yet to be elucidated. Macrophages are directly infected by HIV-1, mediate the dissemination of the virus and contribute to the chronic activation of the inflammatory response observed in infected individuals. Therefore, we characterized the effects of four host mRNA decay proteins, i.e., UPF1, UPF2, SMG6 and Staufen1, on viral replication in HIV-1-infected primary monocyte-derived macrophages (MDMs).

**Results:**

Steady-state expression levels of these host mRNA decay proteins were significantly downregulated in HIV-1-infected MDMs. Moreover, UPF2 and SMG6 inhibited HIV-1 gene expression in macrophages to a similar level achieved by SAMHD1, by directly influencing viral genomic RNA levels. Staufen1, a host protein also involved in UPF1-dependent mRNA decay and that acts at several HIV-1 replication steps, enhanced HIV-1 gene expression in MDMs.

**Conclusions:**

These results provide new evidence for roles of host mRNA decay proteins in regulating HIV-1 replication in infected macrophages and can serve as potential targets for broad-spectrum antiviral therapeutics.

**Electronic supplementary material:**

The online version of this article (10.1186/s12977-019-0465-2) contains supplementary material, which is available to authorized users.

## Background

Macrophages are cells of the myeloid lineage that serve important functions in the host innate immune response. They recognise and phagocytose invading pathogens and play many roles in tissue development, homeostasis and repair [1]. They are present in most tissues in the body and arise from the terminal differentiation of infiltrating monocytes [2]. Examples of tissue-resident macrophages are the alveolar macrophages in the lung, Kupffer cells in the liver and the microglial cells of the central nervous system [3].

Macrophages play multiple roles in human immunodeficiency virus type 1 (HIV-1) pathogenesis (reviewed in [4–6]) as they express the host cell surface receptors CD4 and CCR5 required for HIV-1 entry and thus, can be directly infected by HIV-1 [7, 8]. They promote the dissemination and cell-to-cell transmission of HIV-1 via virological synapses [9–11] and they can also be infected *in trans* by the selective capture and engulfment of HIV-1-infected CD4^+^ T cells [12]. Furthermore, they directly contribute to pathogenesis via the activation of inflammatory pathways resulting in the cognitive dysfunction, respiratory dysfunction, cardiovascular disease and microbial translocation in the intestine associated with HIV-1 infection (reviewed in [5]).

The ability of HIV-1 to rapidly form a stable viral reservoir upon infection is the major obstacle towards an HIV-1 cure [13]. Most studies on HIV-1 latency have focused on CD4+ T cells. However, the contribution of cells of the myeloid lineage to the maintenance of HIV-1 latency has recently been recognised [14]. Macrophages have been proposed to represent a long-lived HIV-1 viral reservoir [5, 15–17], as they have a longer life-span than CD4+ T cells and possess self-renewing properties [18]. During HIV-1 infection, macrophages are more resistant to the cytopathic effects of the virus and display increased telomerase activity which contributes to their increased longevity [19, 20]. In in vivo studies using humanised mouse models, tissue-resident macrophages sustain and propagate HIV-1 infection independently of CD4+ T cells [21]. In follow-up studies using the same humanized myeloid-only mouse model, HIV-1 infection was rapidly suppressed by combination antiretroviral treatment (cART) and viral rebound was observed in a third of the mice following the discontinuation of cART, thus representing the first direct evidence of HIV-1 persistence in tissue macrophages in vivo [17]. Moreover, macrophages were also demonstrated to function as a latent reservoir in SIV-infected, ART-treated macaques [22].

One of the strategies to cure HIV-1 infection is the “kick and kill” approach. This strategy involves the use of latency-reversing agents (LRAs) to stimulate virus production from latently-infected cells; followed by their elimination by the host immune system, cytopathic effects of virus production or cART [23]. These LRAs induce viral production in CD4+ T cells [24]. However, LRA treatment in macrophages resulted in decreased viral release due to the activation of autophagy by the LRAs and the degradation of intracellular viral proteins [25]. Moreover, in a study evaluating the efficacy of a combination of two LRAs (i.e. byrostatin and JQ1), latent proviruses were more efficiently reactivated in monocytic cells that in lymphoid cells [26]. These results highlighted differential responses to LRAs that exist between T cells and macrophages during HIV-1 infection. Therefore, a greater understanding for the roles of host cell proteins that control HIV-1 gene expression in macrophages is needed.

mRNA surveillance pathways are host quality control mechanisms that degrade aberrant mRNA to prevent the accumulation of potentially toxic truncated or misfolded proteins. Examples of these pathways include the nonsense-mediated mRNA decay (NMD) and Staufen1-mediated decay (SMD) [27, 28]. Up-frameshift protein 1 (UPF1), the central player in these mechanisms, is a multifunctional RNA-binding protein that has ATPase and RNA helicase activity [29]. During the process of NMD, UPF1 interacts with a family of up-frameshift proteins such as UPF2, UPF3A and UPF3B, and its associated suppressor for morphological defects in genitalia (SMG) proteins such as a kinase SMG1, an endonuclease SMG6, SMG5 and SMG7, resulting in the degradation of aberrant mRNAs (reviewed in [30, 31]). UPF1 is also involved in the mRNA decay process of SMD that is mediated by the host protein Staufen1 [32].

In our earlier studies, we demonstrated that the HIV-1 genomic RNA (vRNA) is able to evade mRNA surveillance in HeLa cells and CD4^+^ T cells [33–35]. Moreover, HIV-1 hijacks UPF1 and Staufen1 to promote vRNA stability and ensure viral gene expression and production of the main HIV-1 structural protein, pr55^Gag^. The vRNA assembles into an HIV-1-dependent ribonucleoprotein complex (RNP) with UPF1 and Staufen1, resulting in enhanced vRNA stability, nucleocytoplasmic export and translation [33, 36]. UPF2 is excluded from this RNP and is detrimental to the nucleocytoplasmic export of the vRNA, with an overexpression of UPF2 resulting in nuclear sequestration of the vRNA and reduced expression of pr55^Gag^ [34]. UPF1 also has characterised roles in facilitating HIV-1 reverse transcription [37]. In CD4^+^ T cells, both SMG6, the endonuclease involved in the final step of the degradation of aberrant RNA in NMD, and UPF2 are detrimental to vRNA expression in a UPF1-dependent manner [35]. Staufen1 also plays roles at various steps of virus replication including pr55^Gag^ multimerisation, vRNA encapsidation [38–42] and, more recently, in countering host stress responses [43].

In this study, we have characterised the effects of UPF1, UPF2, SMG6 and Staufen1 on viral replication in primary monocyte-derived macrophages (MDMs). We observed that these proteins had significant effects on HIV-1 replication in MDMs. The identification of novel host proteins capable of restricting HIV-1 replication in MDMs will pave the way for novel targets for therapeutic intervention.

## Results

### Relative protein expression levels for UPF1, UPF2 and SMG6 are decreased in HIV-1-infected MDMs

**Fig. 1:**
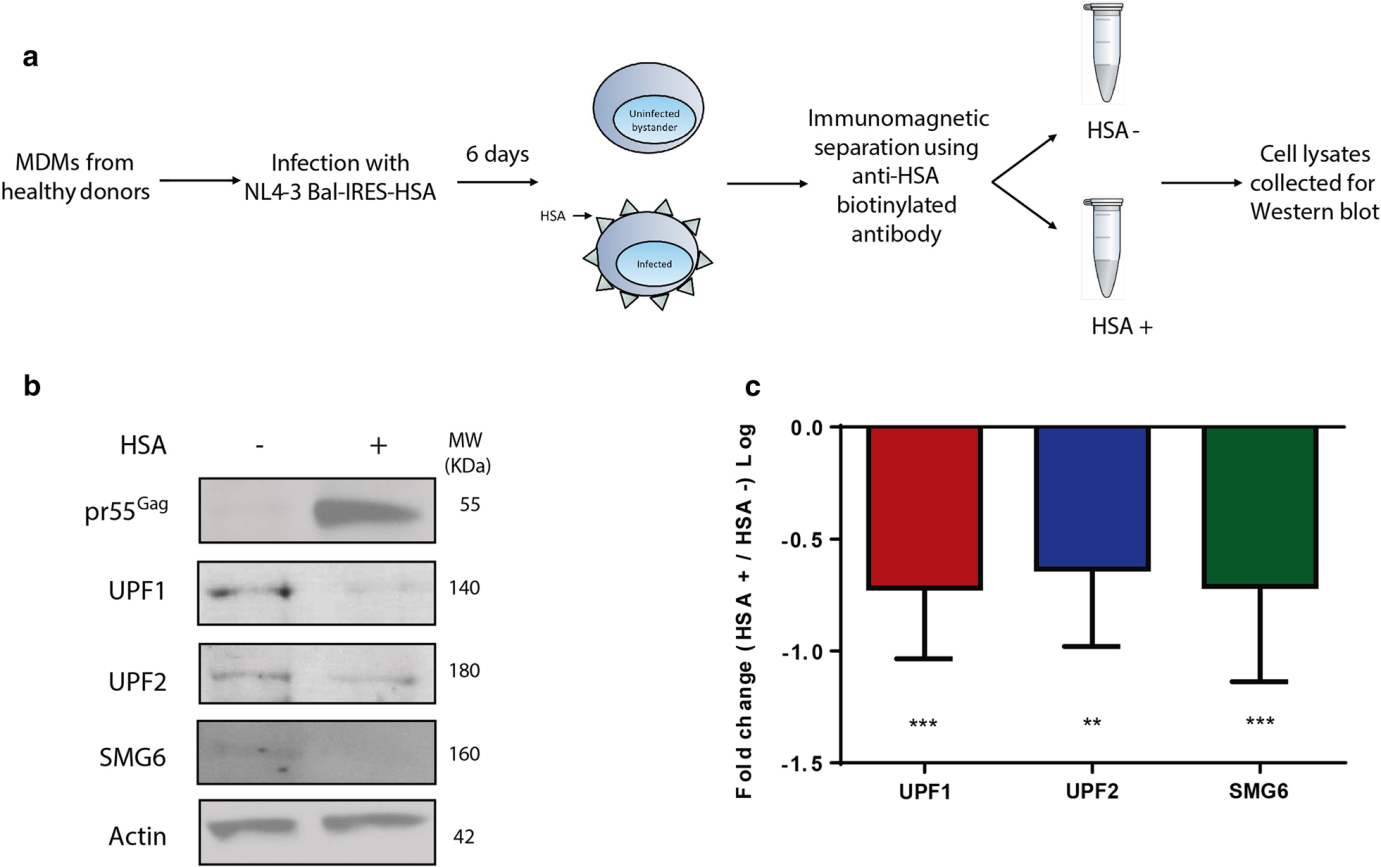
UPF1, UPF2, and SMG6 expression are reduced in HIV-1-infected MDMs. Human monocytes were differentiated into MDMs and infected with NL4.3-Bal-IRES-HSA virus (MOI: 1.0). Cells were collected at 6 days post-infection, incubated with anti-HSA antibody and sorted through magnetic separation as described previously [44]. **a** Schematic of the sorting strategy to separate HSA-negative from HSA-positive cells. **b** Cell lysates were run on SDS-PAGE gels and UPF1, UPF2, SMG6, pr55^Gag^ and actin protein levels were detected by Western Blotting. **c** Fold changes in expression levels of each protein between bystander and HIV-1-infected cells. Error bars represent the standard deviation from three independent experiments with cells from three different donors each. Asterisks represent statistically significant difference between bystander and infected cells (One-way ANOVA; **p ≤ 0.01 and *p* ***p ≤ 0.001)

Previous work from our group has demonstrated that the NMD proteins UPF1, UPF2 and SMG6 have differential effects on vRNA metabolism in cells of the lymphoid lineage [33–35]. Therefore, we hypothesized that RNA surveillance proteins can also impact HIV-1 gene expression in primary MDMs. To determine whether the expression of these proteins is modulated during HIV-1 infection, we assessed the levels of UPF1, UPF2 and SMG6 expression in HIV-1-infected primary MDMs using an HIV-1 reporter construct called NL4-3-Bal-IRES-HSA [44–46]. This an infectious molecular clone of HIV-1 that can infect myeloid cells including human monocyte-derived macrophages and expresses the gp160 HIV-1 envelope of the *Env* gene of the R5-tropic HIV-1 Bal and all other viral genes of the T-cell tropic HIV-1 isolate NL4.3 [44]. Additionally, this viral construct also expresses a murine heat-stable antigen (HSA), a cell surface reporter that allows the detection of cells that are productively infected with HIV-1 [46]. All the data presented in this manuscript were generated using MDMs from at least three donors, unless indicated otherwise. After 3 days of resting post-differentiation, MDMs were infected with NL4-3-Bal-IRES-HSA virus. Cells were collected 6 days post-infection, incubated with anti-HSA antibody and sorted through magnetic separation as depicted in Fig. 1a and described in a previous paper [44]. Whole-cell lysates were collected from HSA-positive (HIV-1-infected cells) and HSA-negative (bystander cells), and the expression levels of UPF1, UPF2 and SMG6 were quantified by Western blotting. As expected, pr55^Gag^ expression was detected only in the HSA-positive populations, indicating that HIV-1 infected and bystander cells were efficiently separated (Fig. 1b). Importantly, significantly lower expression of the NMD proteins UPF1, UPF2 and SMG6 were detected in HIV-1-infected MDMs (Fig. 1b, c), with a decrease of 0.71 (± 0.09) log for UPF1, 0.63 (± 0.10) log for UPF2 and 0.71 (± 0.15) log for SMG6. These findings indicate that the expression levels of these NMD proteins are either downregulated during HIV-1 replication, or that the population with higher expression of the NMD proteins is refractory to productive HIV-1 infection. Therefore, our results suggest that these proteins may play a role in the HIV-1 life cycle.

### UPF2 and SMG6 restrict HIV-1 replication and viral gene expression in primary MDMs

**Fig. 2:**
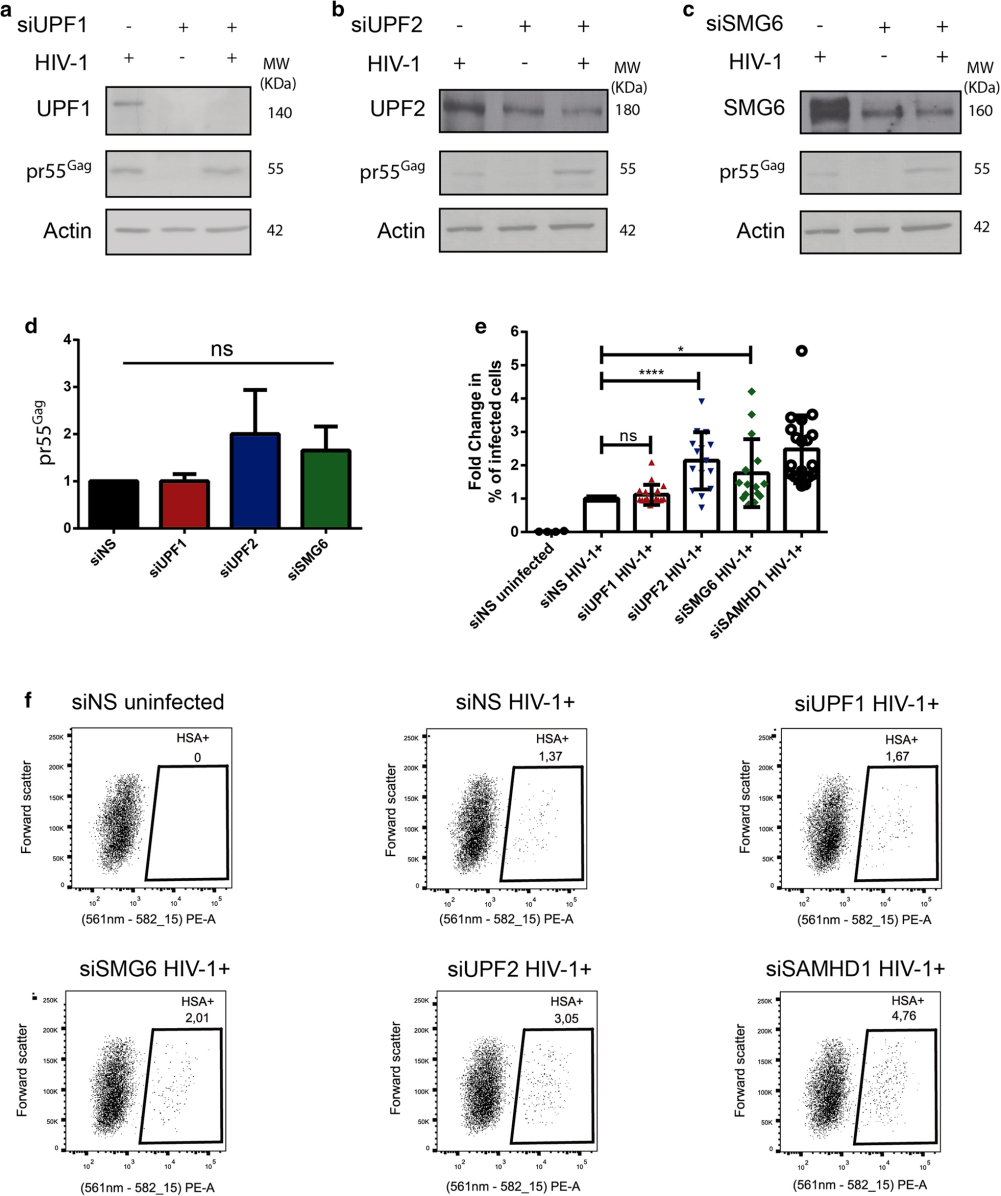
UPF2 and SMG6 knockdown enhance HIV-1 viral gene expression and replication in primary MDMs. Human monocytes were differentiated into MDMs and then transfected with the indicated siRNAs. After 24 h, cells were infected with NL4-3-Bal-IRES-HSA virus (MOI: 1.0) and kept in culture for 6 days. Cells silenced for **a** UPF1, **b** UPF2 or **c** SMG6 were collected, lysates were run on SDS-PAGE gels and protein levels were detected by Western blotting. **d** Fold change in the levels of pr55^Gag^ normalized to the siNS condition. Error bars represent the standard deviation from three independent experiments with cells from three different donors each. Asterisks represent statistically significant difference between groups (One-way ANOVA; ns: not significant). **e** Cells silenced for UPF1, UPF2, SMG6 or SAMHD1 were collected, incubated with anti-HSA antibody and analysed by flow cytometry. Fold change in the HSA expression was normalized to the siNS condition. Error bars represent the standard deviation from three independent experiments with cells from 5 different donors each. Asterisks represent statistically significant difference between groups (One-way ANOVA; ns: not significant, *p ≤ 0.05 and *p* ****p ≤ 0.0001). **f** Representative dot plot depicting HSA expression in siNS, siUPF1, siUPF2, siSMG6 and siSAMHD1 transfected primary MDMs

Since we observed lower levels of UPF1, UPF2 and SMG6 in HIV-1-infected primary MDMs, we performed siRNA-mediated depletion of these NMD proteins in primary MDMs and evaluated the overall effect on HIV-1 gene expression. Cells were either transfected with a non-silencing siRNA (siNS) or with siRNA directed against UPF1 (siUPF1), UPF2 (siUPF2) or SMG6 (siSMG6). On the following day, cells were infected with NL4-3-Bal-IRES-HSA virus. siRNA-mediated silencing was repeated 2 days after infection to maintain gene knockdown. Cells were collected 6 days post-infection and whole cell lysates were analysed by Western blotting. Following siRNA transfection, UPF1, UPF2 and SMG6 expression were efficiently reduced by at least 70% in all cases (Fig. 2a–c, Additional file 1: Fig. S1A–D). Interestingly, we did not observe any significant change in pr55^Gag^ levels in cells transfected with siUPF1 (Fig. 2a, d). However, 2.00 (± 0.93) and 1.65 (± 0.51)-fold increase in pr55^Gag^ levels was observed in cells silenced for UPF2 and SMG6, respectively (Fig. 2b–d). Although these values are statistically insignificant (*p* = 0.1363 for siUPF2 and *p* = 0.0916 for siSMG6 condition), and a trend towards increased pr55^Gag^ levels upon knockdown of UPF2 and SMG6 in cells was observed suggesting that these proteins are detrimental for HIV-1 replication in MDMs.

To further quantify the effect of UPF1, UPF2 and SMG6 on the ability of cells to be productively infected by HIV-1, cells were treated with the siRNAs as described above and the percentage of infected cells were monitored by flow cytometry using antibodies against the HSA tag. An siRNA against SAMHD1 (siSAMHD1) was used as a positive control. SAMHD1 is a well-characterized HIV-1 restriction factor in macrophages and we expect that the MDMs depleted of SAMHD1 are more permissive to productive HIV-1 infection [47]. The experiments were conducted on MDMs from 5 independent donors in triplicate. Consistent with the effects observed on pr55^Gag^ levels by Western blotting (Fig. 2a–d), no significant difference was found in the percentage of infected cells between siNS and siUPF1 transfected cells (Fig. 2e, f). However, a statistically significant and striking increase in the percentage of infected cells was observed in cells depleted of UPF2, with a 2.14 (± 0.85)- fold increase in the percentage of cells productively-infected with HIV-1 when compared to that found in the siNS condition (Fig. 2e, f). This increase in HIV-1 replication is comparable to cells transfected with siSAMHD1 that exhibited a 2.48 (± 1.01)- fold increase in the percentage of productively-infected cells (Fig. 2e, f). A knockdown of SMG6 resulted in a 1.77 (± 1.02)- fold increase in the percentage of infected cells as compared to the siNS condition. In order to determine if the increase in pr55^Gag^ levels observed in Fig. 2e is due to enhanced translation of the vRNA or due to increased number of productively infected cells, we normalised the values from Fig. 2e to the % of productively infected cells from Fig. 2f and observed no significant differences in pr55^Gag^ levels upon NMD protein knockdowns after normalisation (Additional file 1: Fig. S2A). This implies that the NMD proteins are not enhancing vRNA translation and that the increased pr55^Gag^ observed in Fig. 2e is a result of increased number of productively infected cells observed in Fig. 2f. These results are consistent with our previous observations that UPF2 and SMG6 are detrimental to vRNA levels [34]. The results herein indicate that UPF2 and SMG6 impair productive HIV-1 replication in primary MDMs and inhibit vRNA expression.

### UPF2 and SMG6 downregulate vRNA levels in primary HIV-1 infected MDMs

**Fig. 3:**
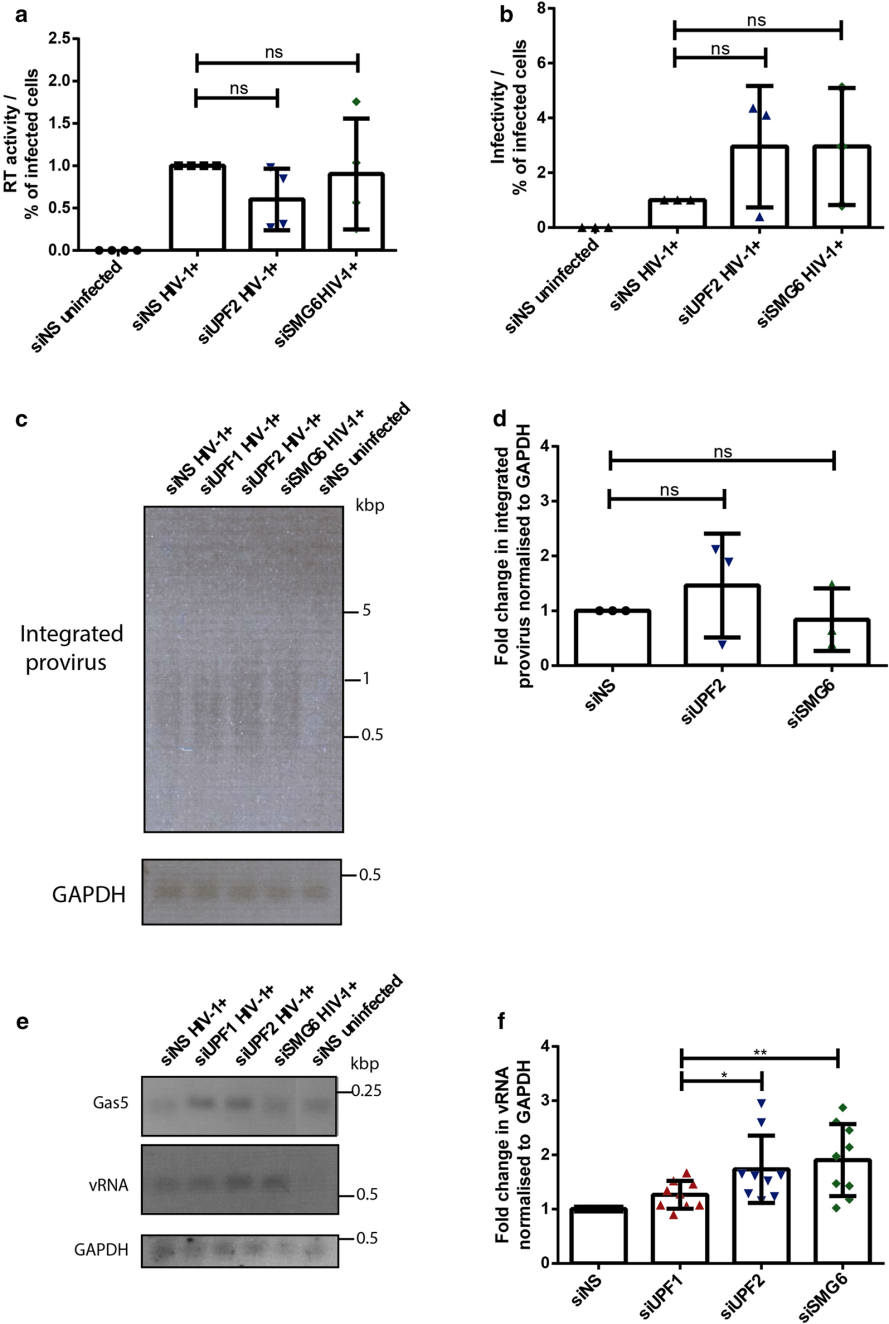
UPF2 and SMG6 knockdown enhance HIV-1 vRNA expression in primary HIV-1-infected MDMs. Human monocytes were differentiated into MDMs and then transfected with control siRNA (siNS) or siRNAs directed against UPF1, UPF2 or SMG6. After 24 h, cells were infected with NL4-3-Bal-IRES-HSA virus (MOI: 1.0) and kept in culture for 6 days. **a** RT activity in cell supernatants was analysed and fold changes in the RT activity were normalized to the siNS condition and to the % of infected cells in each condition. Error bars represent the standard deviation from three independent experiments with cells from three different donors each (One-way ANOVA; ns: not significant). **b** Viral titre in cell supernatants was quantified using the X-gal staining assay in TZM-bl cells and fold changes in viral titre were normalized to the siNS condition and to the % of infected cells in each condition. Error bars represent the standard deviation from three independent experiments with cells from three different donors each (One-way ANOVA; ns: not significant). **c** Integrated proviral DNA was measured using a combined Alu-HIV-1 PCR and PCR products were visualized in a 1% agarose gel and DNA staining **d** Fold change in the levels of integrated proviral DNA visualized in C and normalized to the siNS condition. Error bars represent the standard deviation from three independent experiments with cells from three different donors each (One-way ANOVA; ns: not significant). **e** The NMD target Gas5 mRNA and vRNA levels were measured by RT-PCR and PCR products were visualized on a 1% agarose gel and DNA staining. **f** Fold change in the levels of vRNA visualized in E and normalized to the siNS HIV-1 + condition. Error bars represent the standard deviation from three independent experiments with cells from three different donors each (One-way ANOVA; ns: not significant, *p ≤ 0.05 and *p* **p ≤ 0.01)

Since the silencing of UPF2 and SMG6 led to higher levels of intracellular pr55^Gag^ and increased percentages of productively infected cells, we next sought to determine the stage of viral replication where UPF2 and SMG6 inhibit viral replication. We first validated that the virus being produced from UPF2- and SMG6- depleted cells were not defective. Primary MDMs were transfected with control siRNAs (siNS) or siRNAs against UPF2 and SMG6 and infected with NL4-3-Bal-IRES-HSA virus as described above. At 6 days post infection, virus production was quantified by the reverse transcriptase (RT) activity in the cell supernatant and the results were normalized to the percentage of infected cells in each condition. We observed no statistically significant differences between the treatments (Fig. 3a). To determine if the silencing of these proteins has any effect on the infectivity of the viral progeny, we also measured the production of infectious viral particles in the supernatant of each condition using an X-gal staining assay in TZM-bl cells as described previously [35, 48]. The infectivity was calculated per volume of the supernatant. When normalised to the percentage of infected cells, increased (albeit statistically insignificant) numbers of virus particles were released with UPF2 and SMG6 knockdown, possibly due to increased number of productively infected cells (Fig. 3b). When the number of infectious virus particles was normalised to the RT activity of the virus from the supernatant, increased (albeit statistically insignificant) increase in viral infectivity was observed upon UPF2 and SMG6 knockdown (Additional file 1: Fig. S2A). These findings suggest that the virus produced from MDMs depleted of the NMD proteins UPF2 and SMG6 are not defective in the late stages of viral replication (i.e., budding and maturation) and may even have increased infectivity.

We then distinguished if the effects of UPF2 and SMG6 on HIV-1 replication were observed at a stage before the integration of the proviral DNA into the host genome or at a post-integration stage. Proviral DNA integration in control and UPF1-, UPF2- and SMG6-silenced MDMs was measured using a combined Alu-HIV-1 PCR as described in [49]. We observed no statistically significant differences between the amounts of integrated provirus across all conditions described (Fig. 3c, d). This suggests that UPF2 and SMG6 inhibit HIV-1 replication in primary MDMs at a post-integration stage.

**Fig. 4:**
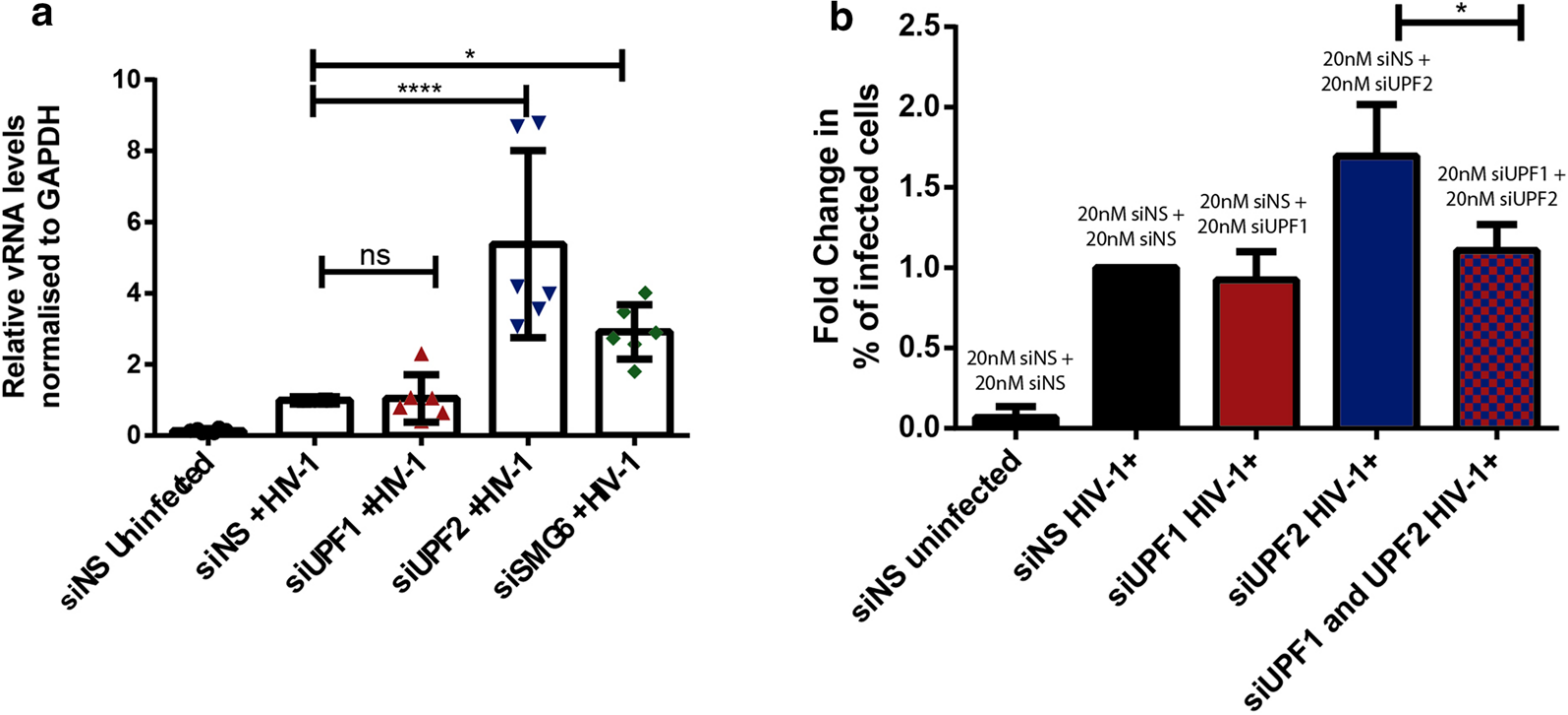
UPF2 and SMG6 knockdown enhance HIV-1 vRNA expression in primary HIV-1-infected MDMs. Human monocytes were differentiated into MDMs and then transfected with control siRNA (siNS) or siRNAs directed against UPF1, UPF2 or SMG6. After 24 h, cells were infected with NL4-3-Bal-IRES-HSA virus (MOI: 1.0) and kept in culture for 6 days. **a** Fold change in the levels of vRNA as measured by RT-qPCR and normalized to the siNS HIV-1 + condition. Error bars represent the standard deviation from two independent experiments with cells from three different donors each (One-way ANOVA; ns: not significant, *p ≤ 0.05 and *p* ****p ≤ 0.0001). **b** Cells were transfected with siNS, siUPF1, siUPF1 or siUPF1 and siUPF2 combined, infected and after 6 days were collected, incubated with anti-HSA antibody and analysed by flow cytometry. Fold change in the HSA expression was normalized to the siNS condition. Error bars represent the standard deviation from three independent experiments with cells from one donor. Asterisks represent statistically significant difference between groups (One-way ANOVA; *p ≤ 0.05)

The NMD proteins are known to directly influence mRNA levels [50]. Thus, we evaluated if UPF2 and SMG6 could also affect intracellular vRNA expression. We also tested if NMD was inhibited upon siRNA-mediated knockdown of the NMD proteins UPF1, UPF2 and SMG6. In MDMs transfected with siNS, siUPF1, siUPF2 and siSMG6, the levels of Gas5 mRNA, which is normally subjected to NMD, were measured by semi-quantitative RT-PCR [51]. Intracellular vRNA expression in each condition was also quantified as described in [34, 38]. Gas5 mRNA levels were increased upon knockdown of UPF1 and UPF2, indicating that NMD is inhibited upon depletion of these proteins (Fig. 3e and Additional file 1: Fig. S2B). Although a modest increase in Gas5 mRNA was observed upon SMG6 knockdown, the levels were not comparable to the increase observed upon UPF1 and UPF2 knockdown (Fig. 3e and Additional file 1: Fig. S2B). This could be because although SMG6 is involved in the degradation of aberrant mRNA during NMD, the mRNA could also be degraded via an SMG6-independent pathway involving the proteins SMG5 and SMG7 [27, 52]. No statistically significant difference was observed in the vRNA levels of cells silenced for UPF1 as compared to control cells (Fig. 3e, f). However, the MDMs depleted of UPF2 and SMG6 presented a 1.74 (± 0.62)- and 1.91 (± 0.66)- fold increase in the expression of intracellular genomic, unspliced vRNA (Fig. 3e, f). To further validate the direct effect of UPF2 and SMG6 on vRNA levels in HIV-1 infected macrophages, we conducted RT-qPCR to measure intracellular vRNA in siNS, siUPF1, siUPF2 and siSMG6 treated macrophages. A knockdown of UPF2 and SMG6 resulted in a 5.38 (± 2.6) and a 2.92 (± 0.76) fold increase respectively in intracellular genomic vRNA levels (Fig. 4a). These results suggest that the NMD proteins UPF2 and SMG6 inhibit viral gene expression by directly influencing vRNA levels at a post-integration stage of the HIV-1 life cycle.

UPF2 is known to bind UPF1 with a high affinity [53]. Additionally, previous work from our research group has demonstrated that the detrimental effects of UPF2 on vRNA metabolism are directly related to its binding to UPF1 [34]. Since the silencing of UPF2 led to an increased percentage of infected cells and intracellular pr55^Gag^ in primary MDMs (Fig. 2d, e), we next determined whether this effect was dependent on UPF1. We transfected primary MDMs from one donor in three independent experiments with control siRNA (siNS), siUPF1 or siUPF2 alone or siUPF1 and siUPF2 combined (20 nM of each siRNA per condition, 40 nM in total) and quantified the percentage of infected cells by detecting the expression of the HSA tag by flow cytometry. We observed that, in the doubly-silenced cells, the proportion of productively infected cells is comparable to control cells (Fig. 4b), indicating that the deleterious effect of UPF2 on viral replication in primary MDMs depends on UPF1.

### Staufen1 enhances HIV-1 gene expression in primary MDMs

**Fig. 5:**
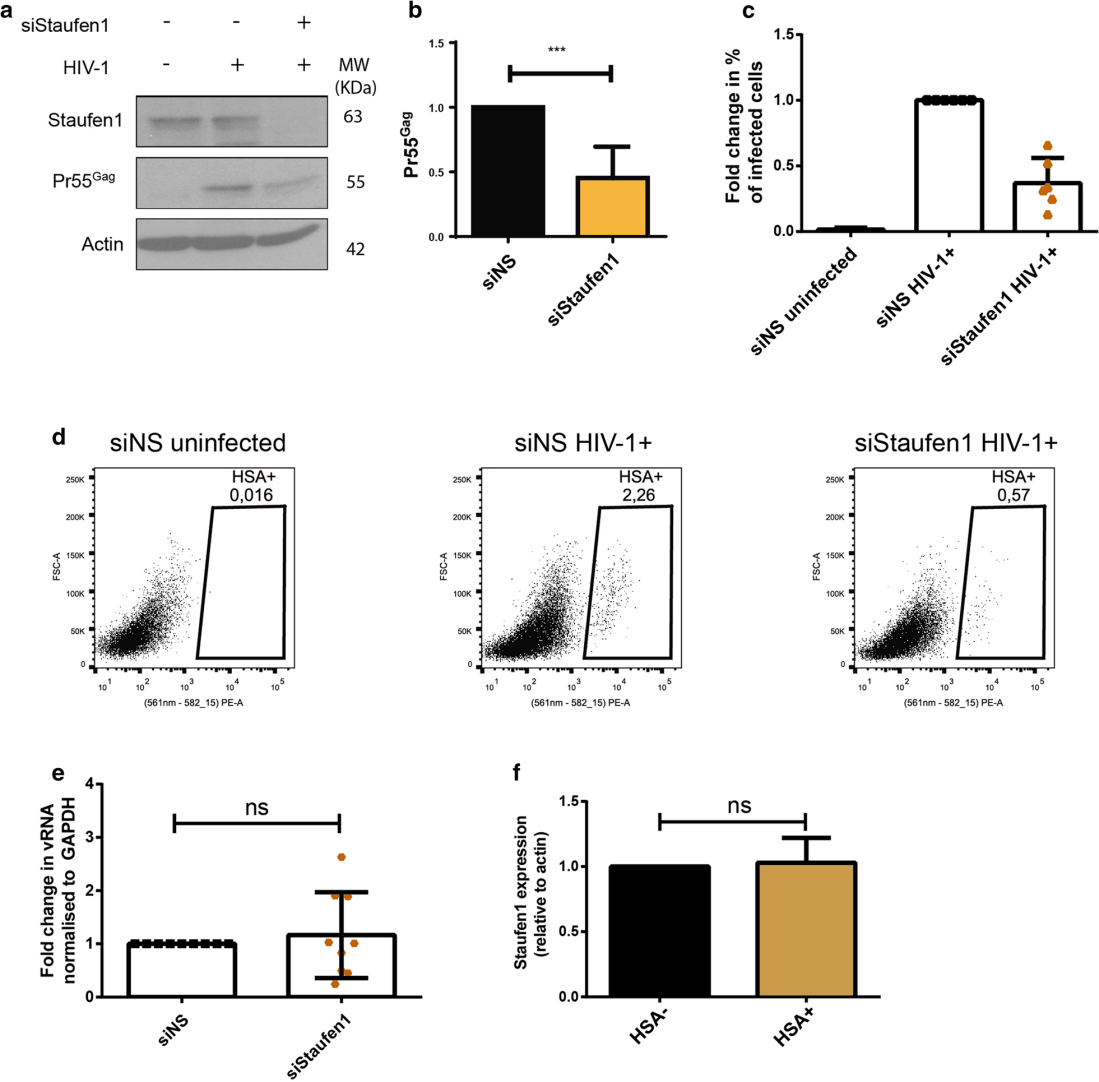
Staufen1 knockdown impairs HIV-1 viral gene expression and replication in primary HIV-1-infected MDMs. Human monocytes were differentiated into MDMs and then transfected with control siRNA (siNS) or siRNA against Staufen1. After 24 h, cells were infected with NL4-3-Bal-IRES-HSA virus (MOI: 1.0) and kept in culture for 6 days. **a** Cells were collected, lysates were run on SDS-PAGE gels and protein levels were detected by Western Blotting. **b** Fold change in the levels of pr55^Gag^ normalized to the siNS condition. Error bars represent the standard deviation from three independent experiments with cells from three different donors each (One-way ANOVA; p ***p ≤ 0.001). **c** Cells were collected, incubated with anti-HSA antibody and analysed by flow cytometry. Fold change in the HSA expression was normalized to the siNS condition. Error bars represent the standard deviation from three independent experiments with cells from three different donors each. **d** Representative dot plot depicting HSA expression in siNS and siStaufen1 transfected primary MDMs. **e** vRNA was measured by RT-PCR and fold change in the levels of vRNA were normalized to the siNS condition. Error bars represent the standard deviation from three independent experiments with cells from three different donors each (One-way ANOVA; ns: not significant). **f** Cells were collected at 6 days post-infection, incubated with anti-HSA antibody and sorted through magnetic separation as described in Fig. 1a. Cell lysates were run on SDS-PAGE gels and Staufen1 and actin protein levels were detected by Western Blotting and the fold changes in expression levels between bystander (HSA−) and HIV-1-infected cells (HSA+) were quantified. Error bars represent the standard deviation from two independent experiments with cells from two different donors each. (Student’s *t* test; *ns* not significant)

In addition to NMD, mammalian cells harbour another UPF1-dependent RNA surveillance pathway called Staufen1-mediated decay (SMD), in which the mRNA degradation process is mediated by the binding of Staufen1 to the 3′-untranslated region (3′-UTR) of target mRNAs and the subsequent recruitment of UPF1 (reviewed in [28]). Staufen1 has been previously demonstrated to bind to the vRNA in the cytoplasm, facilitate translation initiation of the vRNA and be selectively packaged into HIV-1 virions [39, 42, 54]. Therefore, we next sought to determine if these effects of Staufen1 on HIV-1 replication are also observed in primary MDMs. Cells were either transfected with a non-silencing siRNA (siNS) or with siRNA against Staufen1 (siStaufen1) and subsequently infected with NL4-3-Bal-IRES-HSA virus. Silencing was repeated 2 days after infection to maintain gene knockdown. Cells lysates collected 6 days post-infection and analysed by Western blotting and the percentage of infected cells was monitored by detection of the HSA tag by flow cytometry. We observed that silencing of Staufen1 led to a significant decrease in intracellular pr55^Gag^ (54.7 ± 0.1%) (Fig. 5a, b). The knockdown of Staufen1 also resulted in a 63.02 (± 19.05) % decrease in the percentage of infected cells as compared to the mock-treated cells (Fig. 5c, d). In order to determine if this effect was due to a reduction in vRNA stability or a defect in vRNA translation, we conducted semi-quantitative RT-PCR from whole cell lysates in the above described conditions. No significant difference in the intracellular levels of vRNA between siNS and siStaufen1-transfected cells was observed (Fig. 5e). We also determined if HIV-1 infection in MDMs results in differential expression of Staufen1 by HSA magnetic separation technique described in the Fig. 1a and observed no significant difference in Staufen1 levels in HIV-1 infected and bystander cells (Fig. 5f). Overall, our data suggests that Staufen1 enhances the translation of the vRNA in primary MDMs, similarly to previous data that was observed in other cell types [38, 42].

## Discussion

Cellular mRNA quality control machineries are a conserved form of intrinsic antiviral immunity [55–57]. In our work, we demonstrate that a depletion of the NMD proteins UPF2 and SMG6 result in increased levels of HIV-1 vRNA expression and viral production (Figs. 2b–d, 3e, f). This antagonistic effect of NMD on viral infections in mammalian cells is also highlighted in recent reports that demonstrate that the NMD proteins UPF1, SMG5 and SMG7 restrict the replication of Semliki Forest virus (SFV) and Sindbis virus of the Togaviridae family and the genomic RNA of SFV was found to be a substrate for NMD, with UPF1 depletion resulting in a nearly 20-fold increase in virus production [51, 56, 58]. In order to ensure viral gene expression, members of the *Retroviridae* family such as Rous sarcoma virus (RSV) and human T-lymphotropic virus type 1 (HTLV-1) have evolved mechanisms to evade NMD [59–63].The full-length unspliced transcript of the retrovirus Rous sarcoma virus (RSV) contains a PTC in the Gag ORF but evades NMD by virtue of the existence of a cis-acting RNA element termed the RNA stability element (RSE) downstream of the Gag PTC that binds to the polypyrimidine tract binding protein 1 (PTBP1) and prevents the recruitment of UPF1 [59, 64, 65]. Another retrovirus, the human T cell lymphotropic virus-type 1 (HTLV-1), also evades NMD via the action of its two viral proteins Tax and Rex [61–63]. Tax binds UPF1 and inhibits its RNA-binding activity, resulting in partial inhibition of NMD while Rex has an important role in stabilising viral transcripts [63, 66]. Tax also prevents the translocation of UPF1 on mRNA to inhibit NMD [63]. Therefore, in order to prevent the antiviral effect of NMD, viruses have evolved mechanisms to evade RNA quality control and ensure gene expression.

In our previous work, we have demonstrated that HIV-1 also evades NMD and hijacks UPF1 to promote HIV-1 genomic RNA stability, nucleocytoplasmic export and translation [33, 34]. It is important to note that these effects of UPF1 on the vRNA are independent of its function in NMD and the expression of an NMD-null UPF1 construct also resulted in enhanced vRNA stability and translation [33]. UPF1 was also demonstrated to promote vRNA stability and viral gene expression in primary CD4+ T cells [35].

In primary MDMs, the knockdown of UPF1 did not have a significant effect on viral replication. However, the NMD proteins UPF2 and SMG6 inhibited HIV-1 gene expression in primary MDMs by downregulating vRNA levels (Fig. 3e, f). The observation that UPF2 and SMG6 were detrimental to viral gene expression is consistent with our previous work, while the effects of UPF1 are in stark contrast to our earlier work that examines the effects of UPF1, UPF2 and SMG6 on the unspliced genomic vRNA [33–35]. Although the UPF1 has been demonstrated to have no effect on the multiply spliced/singly spliced viral RNA levels in previous work [33], it would be interesting to determine if UPF2 and SMG6 also have an effect on the multiply spliced/singly spliced viral RNA transcripts.

In this study, the method of differentiation of macrophages yielded non-polarised/M0 macrophages [46]. Therefore, the conclusions derived from this work applies to the non-polarised phenotype of macrophages. It must be noted that in our work, we utilised a viral construct that was a hybrid of T-cell and M-cell tropic viruses, with only the Env proteins being M-tropic [44]. Whether the use of a completely M-tropic virus will result in differential effects of these proteins on HIV-1 gene expression in MDMs remains to be elucidated.

UPF2, unlike the other NMD components, has not been associated with non-NMD functions [67]. In cells that were depleted of UPF2, NMD was indeed downregulated, as demonstrated by the increase in the levels of the Gas5 endogenous mRNA targeted by NMD (Fig. 3e). Importantly, the inhibition of NMD by UPF2 knockdown also correlated with an increase in vRNA levels and viral gene expression (Figs. 2e, 3f, 4g). This result implicates a distinct function for NMD in the downregulation of vRNA in MDMs, the second such report of viral RNA being subjected to NMD in mammalian cells [51]. This novel role for NMD in downregulating the vRNA is supported by our observation that the knockdown of SMG6 also resulted in increased vRNA levels and viral gene expression. Moreover, HIV-1-infected MDMs presented statistically significantly lower levels of the NMD proteins UPF1, UPF2 and SMG6 (Fig. 1b, c), indicating that the implication of these host genes in NMD is possibly detrimental to viral gene expression in primary MDMs. Overall, in light of this and our earlier work, we conclude that cell-type differences exist between T cells and MDMs in vRNA metabolism, with the vRNA in HeLa and CD4^+^ T cells being able to evade NMD [33, 35], but not in MDMs.

The question of why UPF1, the central player involved in NMD, did not have an effect on viral replication remains outstanding. We hypothesise that this is due to the multifaceted nature of UPF1. The best characterised role for UPF1 is in NMD. In the context of HIV-1 infection, UPF1 enhanced both vRNA stability and viral gene expression in an NMD- independent manner [33]. On the one hand, since the vRNA is subjected to NMD in MDMs, UPF1-knockdown could have resulted in increased vRNA levels and gene expression due to impaired NMD, as seen in UPF2- and SMG6-depleted MDMs (Fig. 2b–d). On the other hand, the knockdown of UPF1 could also result in reduced levels of UPF1 that stabilises the vRNA in an NMD-independent manner, resulting in little net effect on vRNA in the face of modulating UPF1 expression levels. Therefore, we hypothesise that due to a combined effects of UPF1’s NMD-independent beneficial roles in enhancing vRNA metabolism, and UPF1’s NMD-, UPF2- and SMG6-dependent detrimental effects on vRNA expression, the overall effects of UPF1 knockdown on HIV-1 replication are nullified.

During HIV-1 infection, we have previously demonstrated that UPF2 is excluded from HIV-1 RNPs through antagonistic interactions with the viral or host proteins such as Rev and Staufen1 [34]. The binding of UPF2 to UPF1 induces a conformational change in UPF1 that facilitates its phosphorylation by the kinase SMG1 [68–70]. This conformational change also impairs UPF1’s RNA-binding capacity which could hinder the binding of UPF1 to the vRNA [69]. Furthermore, UPF2 also binds to UPF1 with high affinity [71] and this could limit the availability of UPF1 to bind to the vRNA. In MDMs, a knockdown of UPF2 resulted in increased viral gene expression and we postulate that this is because of two additive mechanisms. Firstly, a reduction in cellular NMD (Fig. 3e) could lead to increased vRNA levels and gene expression (Figs. 2e, 3f). Secondly, a depletion of UPF2 could result in increased levels of hypophosphorylated UPF1 that is capable of binding to and stabilising the vRNA. The results obtained when both UPF1 and UPF2 were depleted did not have any significant effect on viral gene expression, indicating that the effect of UPF2 on downregulating vRNA is entirely UPF1-dependent (Fig. 4a). Since the binding of UPF2 to UPF1 facilitates the phosphorylation of UPF1 by SMG1 [68–70], it would be interesting to further characterise the contribution of the effect of this post-translational modification of UPF1 on the differential regulation of RNA quality control pathways in T cells and macrophages and its subsequent effect on HIV-1 gene expression.

Staufen1 also plays a role in the vRNA metabolism and viral gene expression in primary MDMs, most likely by the assembly of a distinct HIV-1 RNP in the cytoplasm with the vRNA, pr55^Gag^ and UPF1 as we and others have shown and is consistent with previous reports [33, 36, 38, 39, 72]. The depletion of Staufen1 in primary MDMs resulted in decreased levels of intracellular pr55^Gag^ and viral gene expression with little change in steady-state vRNA levels (Fig. 5b–e). These observations suggest a role for Staufen1 in translational derepression [38, 42].

The current antiretroviral drugs have different effects in macrophages as compared to T cells (reviewed in [19]). Moreover, in the context of HIV-1 curative therapies, the effect of LRAs in macrophages have not been effectively characterised and may have off-target effects such as the induction of autophagy [25]. The antifungal drug amphotericin B is reported to reactivate HIV-1 in a model cell line for the HIV-1 latency in macrophages, but not in T lymphocytes, highlighting yet another example of how reactivation from latency is different in T cells and macrophages. Therefore, it is imperative to address these differences when designing novel therapeutics to treat HIV-1 infection.

## Conclusions

In this work, we identified novel targets to modulate HIV-1 gene expression in macrophages. For example, novel small molecule compounds can be used to mimic the activities of UPF2 and SMG6 to impair viral gene expression by binding to UPF1 and subsequently activating the NMD pathway to downregulate vRNA levels. The binding of Staufen1 to the vRNA can also be hindered using vRNA mimics or small molecules to prevent HIV-1 gene expression, similar to the compounds that inhibit the binding of pr55^Gag^ and the viral RNA packaging signal (psi or Ψ) [73]. These strategies would lead to the development of novel broad-spectrum antiretrovirals or a functional HIV-1 cure. Conversely, novel drugs could be generated to either mimic Staufen1 activity on the vRNA or to block the binding of UPF2 to UPF1, thus paving the way for a novel class of post-transcriptional LRAs that are effective across both lymphoid and myeloid components of the HIV-1 reservoir.

## Materials and methods

### Cell culture

PBMCs were isolated by density-gradient centrifugation using lymphocyte separation medium (Corning). Primary monocytes were isolated from PBMCs by the adherence method and were differentiated into monocyte-derived macrophages (MDMs) in 150 mm dishes (Sarstedt) by incubation at 37 °C and 5% CO_2_ for 3 days in RPMI-1640 culture medium (Life Technologies) supplemented with M-CSF (25 ng/mL) (Sigma-Aldrich) and 10% human AB serum (Sigma-Aldrich). Following this period, culture medium was replaced with fresh culture without M-CSF for additional 3 days, then incubated with Accutase Solution (Sigma-Aldrich) for 60–90 min and detached with a cell scraper and cultured in Ultra Low Attachment dishes (ULA, Corning^®^) for 3 additional days again in absence of M-CSF. In earlier work, we characterized the macrophage population obtained following this protocol of MDM differentiation with a short treatment period with M-CSF and we found that the monocytes are differentiated into uncommitted/nonpolarized macrophages (M0 phenotype) that expressed the pan-macrophage marker CD68 in > 97% of cells [46]. Cells were plated at 5 × 10^5^ cells/mL in 12-well plates (Corning). In each experiment, cells from at least three different donors were used unless otherwise stated. HEK293T cells were purchased from the American Type Culture Collection (ATCC). TZM-bl cells were obtained from NIH AIDS Reference and Reagent Program. Both cells lines were cultured in Dulbecco’s modified Eagle medium (DMEM, Invitrogen) containing 10% fetal bovine serum (HyClone) and 1% penicillin–streptomycin (Invitrogen).

### Antibodies

Mouse anti-p24, was obtained from NIH AIDS Reagents Program; rabbit antisera to UPF1 and UPF2 were generously supplied by Jens Lykke-Andersen (University of California, San Diego, CA, USA) [34]; rabbit anti-EST1A (SMG6) and mouse anti-actin were purchased from Abcam; mouse anti-CD24 (henceforth referred as anti-HSA) biotin conjugated clone M1/69 was purchased from BD Biosciences; mouse anti-CD24 (henceforth referred as anti-HSA) PE conjugated clone M1/69 was purchased from eBioscience; rabbit anti-Staufen1 was produced and purified at the McGill University Cell Imaging and Analysis Network (Montréal, Québec, Canada); mouse anti-SAMHD1 was generously supplied by Dr. Mesplède (McGill University) (Abcam); horseradish peroxidase-conjugated secondary antibodies were purchased from Rockland Immunochemicals.

### Virus production and infection

NL4.3-Bal-IRES-HSA virus particles were prepared by transfection of HEK293T cells with HIV-1 NL4.3-Bal-IRES-HSA encoding plasmid [46] using the JetPrime transfection reagent following manufacturer’s instructions. The supernatants were collected 48 h post-transfection, filtered through a 0.45-μm filter (Pall) and centrifuged at 44,800 r.c.f. for 1 h at 4 °C to pellet the virus. Viruses were resuspended in RPMI and stored at − 80 °C. Viral titre was quantified using the X-gal staining assay in TZM-bl cells as described in [48]. Primary MDMs in RPMI culture medium were infected with an MOI of 1.0 for 2 h at 37 °C and 5% CO_2_. Following infection, culture media was supplemented with human serum (Sigma-Aldrich) at a final concentration of 10%. Cells and virus-containing supernatants were collected 6 days post infection.

### Gene silencing

To perform the siRNA transfection in the primary MDMs, 1 μL Lipofectamine 2000 (Life Technologies) was added to 50 μL of RPMI-1640. Each individual siRNA was used at a final concentration of 20 nM for all experiments and diluted in 50 μL of RPMI-1640 into each well of a 12-well cell culture plate. After 20 min of incubation at room temperature, 400 μL of cell suspension containing 5 × 10^5^ cells were added to the mixture containing the Lipofectamine 2000 and siRNAs complexes. Cells were incubated at 37 °C in the presence of 5% CO_2_ for 2 h before adding 500 μL of RPMI-1640 medium supplemented with 20% human serum (10% final concentration). The medium was replaced 24 h after transfection, when infection was performed. Custom siRNA duplexes were synthesised by Qiagen. The target sequence for UPF1 was 5′-AAGATGCAGTTCCGCTCCATT-3′, for UPF2 was 5′-AAGTTGGTACGGGCACTC-3′, for SMG6 was 5′-GCTGCAGGTTACTTACAAG-3′, and for Staufen 1 was 5′-AAATAGCACAGTTTGGAAACT-3 [39]. The siNS used in this study is a commercially available non-silencing control duplex with target sequence 5′-AATTCTCCGAACGTGTCACGT-3′ from Qiagen.

### Cell separation

Cells were separated into virus-infected (HSA +) and uninfected bystander cells (HSA −) using the EasySep Biotin Selection kit (StemCell Technologies) as described in [44, 46]. Briefly, cells were detached by treatment with Accutase Solution (Sigma-Aldrich) for 60 min and washed in DPBS. Next, cells were incubated with the biotinylated anti-HSA antibody biotin-conjugated at a final concentration of 3 μg/mL and separation was performed followed by 5 rounds of magnetic separation of 5 min each in 0.5% BSA.

### Flow cytometry

Flow cytometry analysis was performed with 5 × 10^5^ cells that were incubated anti-HSA PE-conjugated antibody diluted 1:400 in DPBS for 60 min at 37 °C. Cells were then detached by treatment with Accutase Solution (Sigma Aldrich) for 60 min and washed twice in DPBS. Finally, cells were fixed in 4% paraformaldehyde for 30 min and analysed on a BD LSR Fortessa Analyzer. Analysis was performed using the FlowJo V10 software (Treestar).

### Nucleic acid extraction reverse transcription and PCR analysis

Intracellular DNA and RNA extraction were performed using Trizol Reagent (Thermo Fisher Scientific) following manufacturer’s instructions. For RNA samples, cDNA was obtained using the High-Capacity cDNA Reverse Transcription Kit (Applied Biosystems). cDNA and primers were then added to GoTaq Green Master Mix (Promega). GAPDH was amplified using the primers GAPDH_1 forward 5′-TGACCACAGTCCATGCCATC-3′ and GAPDH_1 reverse 5′-ATGATGTTCTGGAGAGCCCC-3′, HIV-1 vRNA using the primers pNL4-3_1 forward 5′-GGGAGCTAGAACGATTCGCA-3′ and pNL4-3_1 reverse 5′-GGATGGTTGTAGCTGTCCCA-3′, and Gas5 using the primers Gas5 forward 5′-GCACCTTATGGACAGTTG-3′ and Gas5 reverse 5′‐GGAGCAGAACCATTAAGC‐3′. For the RT-qPCR, transcript abundance was determined by qPCR using SsoAdvanced SYBR Green supermix (1725270, Bio-Rad). The qPCR analysis was performed in a CFX96 Touch™ Real Time PCR detection system (1855201, Bio-Rad). Data were analyzed by the threshold cycle (Ct) comparative method. Relative expression values were obtained upon normalization to intracellular GAPDH levels. For DNA analysis, DNA and primers were added to the GoTaq Green Master Mix (Promega). GAPDH was amplified using the primers GAPDH_S forward 5′-GCTGATGCCCCCATGTTCGT-3′ and GAPDH_AS reverse 5′-CAAAGGTGGAGGATGGGTGT-3′ and alu-HIV-1-LTR using the primers Alu forward 5′-TCCCAGCTACTCGGGAGGCTGAGG-3′ and M661 reverse 5′-CCTGCGTCGAGAGATCTCCTCTG-3′. The PCR products were visualised on a 1% agarose gel by staining the DNA with RedSafe Nucleic Acid Staining Solution (iNtRON). Signals were captured using a Gel Doc System and intensities were normalised to the GAPDH signal.

### Reverse-transcriptase assay

Reverse transcriptase (RT) activity in cell supernatants was analysed as described previously [74]. Briefly, 5 μL of viral supernatant were added to 50 μL of supplemented RT cocktail and incubated at 37 °C for 2 h. 5 μL of each reaction mixture were spotted onto DEAE filter paper (Whatman). The membranes were washed and read using a Microbeta scintillation counter (PerkinElmer).

### Infectivity assay

Viral titre in cell supernatants was quantified using the X-gal staining assay in TZM-bl cells as described previously [48]. Briefly, different dilutions of supernatants of each condition were added to TZM-bl cells seeded onto 96-well plates (Corning). After 48 h, cells were fixed with 1% paraformaldehyde, washed and treated with X-Gal for the detection of β-galactosidase by counting blue TZM-bl cells.

### Western blotting

Cells were lysed in NP40 lysis buffer (50 mM Tris pH 7.4, 150 mM NaCl, 0.5 mM EDTA, 0.5% NP40). Protein concentration in each cell lysate was quantified by Bradford assay. Equal amounts of protein (20 µg) were separated by SDS-PAGE and transferred to a nitrocellulose membrane (Bio-Rad). Blocking was performed using 5% non-fat milk in Tris-buffered saline (pH 7.4) with 0.1% Tween 20 (TBST) for 1 h at room temperature. Membranes were incubated with the indicated primary and corresponding horseradish peroxidase-conjugated secondary antibodies. Proteins were detected using Western Lightning Plus-ECL (PerkinElmer).

### Statistical analysis

All experiments were performed with at least three donors (unless indicated otherwise) in three independent experiments, and the data are presented as the mean ± standard deviation (SD). A *p* value of < 0.05 in a student’s *t* test, one-way or two-way ANOVA test was considered statistically significant (*p ≤ 0.05, **p ≤ 0.01, ***p ≤ 0.001 and ****p ≤ 0.0001). GraphPad Prism 6 (GraphPad Software Inc.) was used to conduct statistical analyses and create graphs.

## Additional file

**Additional file 1: Figure S1.** Efficiencies of UPF1, UPF2, SMG6 and SAMHD1 knockdowns: Human monocytes were differentiated into MDMs and then transfected with the indicated siRNAs. After 24 h, cells were infected with NL4-3-Bal-IRES-HSA virus (MOI: 1.0) and kept in culture for 6 days. Cells silenced for A) UPF1, B) UPF2, C) SMG6 or D) SAMHD1 were collected, lysates were run on SDS-PAGE gels and protein levels were detected by Western blotting. Fold change in the levels of protein expression normalized to the siNS condition by densitometric analysis. Error bars represent the standard deviation from three independent experiments with cells from three different donors each. **Figure S2.** Effects of UPF1, UPF2 and SMG6 knockdowns: Human monocytes were differentiated into MDMs and then transfected with the indicated siRNAs. After 24 h, cells were infected with NL4-3-Bal-IRES-HSA virus (MOI: 1.0) and kept in culture for 6 days. A) Values from Fig. 2D were normaised to the values from Fig. 2E. B) Viral titre in cell supernatants was quantified using the X-gal staining assay in TZM-bl cells and fold changes in viral titre were normalized to the R activity of virus in the supernatant. Error bars represent the standard deviation from three independent experiments with cells from three different donors each (One-way ANOVA; ns: not significant). C) Fold change in the levels of Gas5 mRNA visualized in Fig. 3E and normalized to the siNS HIV-1 + condition. Error bars represent the standard deviation from three independent experiments with cells from three different donors each (One-way ANOVA; ns: not significant, *p ≤ 0.05 and p **p ≤ 0.01).
