# Supplementary material for: Host mRNA decay proteins influence HIV-1 replication and viral gene expression in primary monocyte-derived macrophages
Source: Retrovirology. 2019 Feb 7;16:3. doi: 10.1186/s12977-019-0465-2 (PMC6367771; doi:10.1186/s12977-019-0465-2)
Supplement: Supplementary file 1 — Additional file 1: Figure S1. Efficiencies of UPF1, UPF2, SMG6 and SAMHD1 knockdowns: Human monocytes were differentiated into MDMs and then transfected with the indicated siRNAs. After 24 h, cells were infected with NL4-3-Bal-IRES-HSA virus (MOI: 1.0) and kept in culture for 6 days. Cells silenced for A) UPF1, B) UPF2, C) SMG6 or D) SAMHD1 were collected, lysates were run on SDS-PAGE gels and protein levels were detected by Western blotting. Fold change in the levels of protein expression normalized to the siNS condition by densitometric analysis. Error bars represent the standard deviation from three independent experiments with cells from three different donors each. Figure S2. Effects of UPF1, UPF2 and SMG6 knockdowns: Human monocytes were differentiated into MDMs and then transfected with the indicated siRNAs. After 24 h, cells were infected with NL4-3-Bal-IRES-HSA virus (MOI: 1.0) and kept in culture for 6 days. A) Values from Fig. 2D were normaised to the values from Fig. 2E. B) Viral titre in cell supernatants was quantified using the X-gal staining assay in TZM-bl cells and fold changes in viral titre were normalized to the R activity of virus in the supernatant. Error bars represent the standard deviation from three independent experiments with cells from three different donors each (One-way ANOVA; ns: not significant). C) Fold change in the levels of Gas5 mRNA visualized in Fig. 3E and normalized to the siNS HIV-1 + condition. Error bars represent the standard deviation from three independent experiments with cells from three different donors each (One-way ANOVA; ns: not significant, *p ≤ 0.05 and p **p ≤ 0.01). [file 12977_2019_465_MOESM1_ESM.pdf]

Supplementary Figure 1

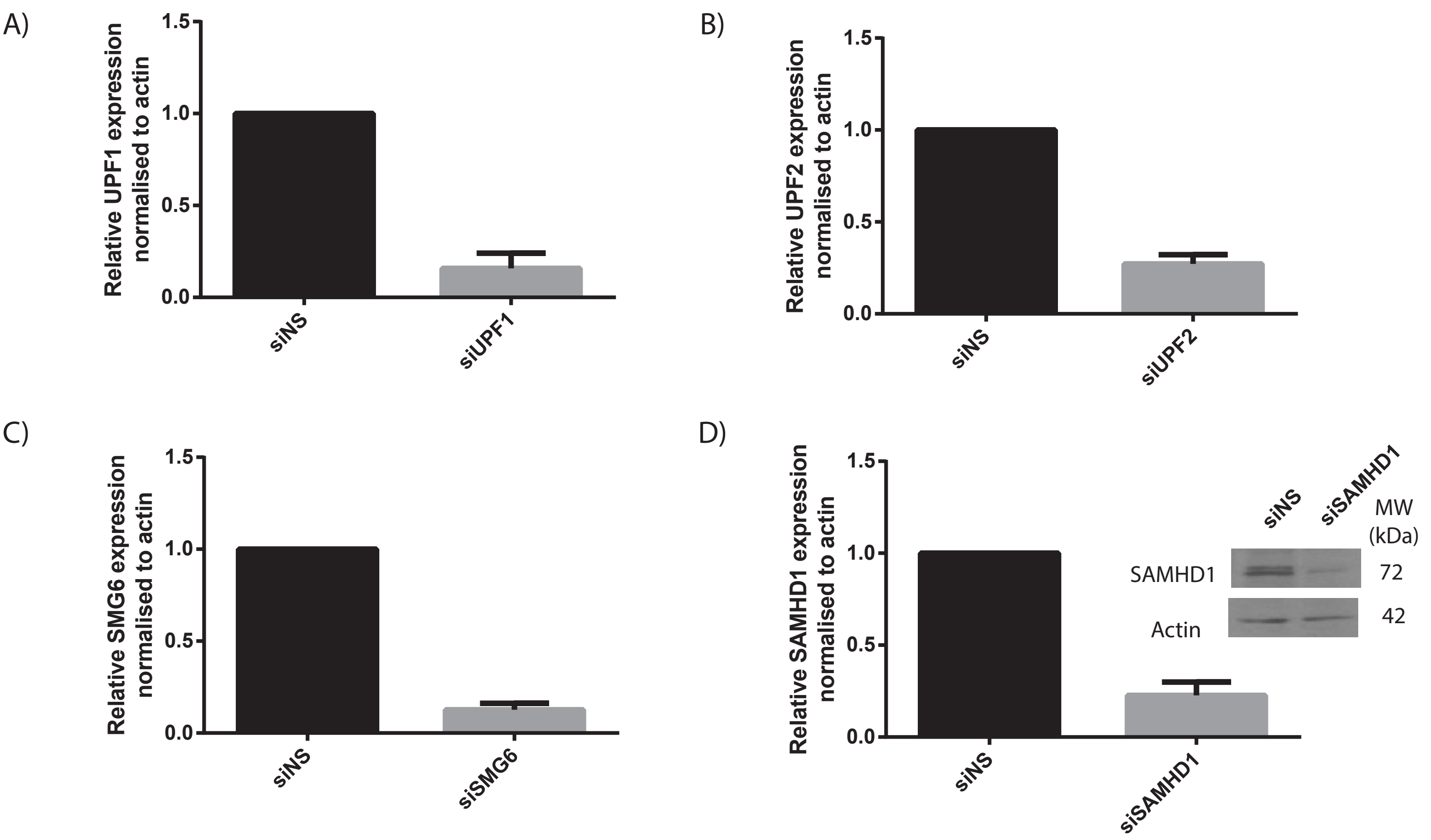

Supplementary Figure 2

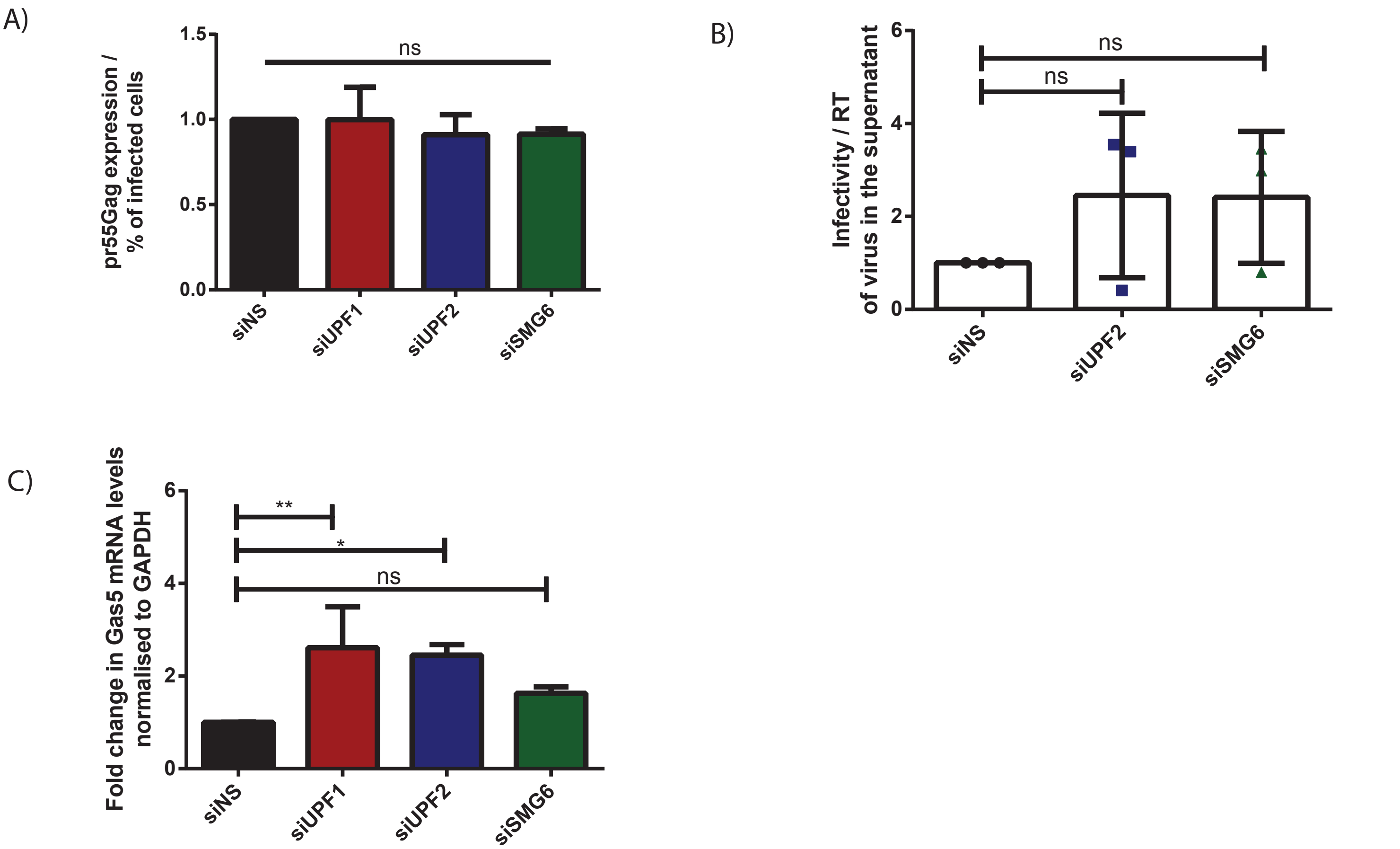

Supplementary figure legends:

Supplementary Figure 1: Effeciencies of UPF1, UPF2, SMG6 and SAMHD1 knockdowns:
Human monocytes were differentiated into MDMs and then transfected with the indicated siRNAs. After 24 h, cells were infected with NL4-3-Bal-IRES-HSA virus (MOI: 1.0) and kept in culture for 6 days. Cells silenced for A) UPF1, B) UPF2, C) SMG6 or D) SAMHD1 were collected, lysates were run on SDS-PAGE gels and protein levels were detected by Western blotting. Fold change in the levels of protein expression normalized to the siNS condition by densitometric analysis. Error bars represent the standard deviation from three independent experiments with cells from three different donors each.

Supplementary figure 2: Effects of UPF1, UPF2 and SMG6 knockdowns:
Human monocytes were differentiated into MDMs and then transfected with the indicated siRNAs. After 24 h, cells were infected with NL4-3-Bal-IRES-HSA virus (MOI: 1.0) and kept in culture for 6 days. A) Values from Figure 2D were normaised to the values from Figure 2E. B) Viral titre in cell supernatants was quantified using the X-gal staining assay in TZM-bl cells and fold changes in viral titre were normalized to the R activity of virus in the supernatant. Error bars represent the standard deviation from three independent experiments with cells from three different donors each (One-way ANOVA; ns: not significant).C) Fold change in the levels of Gas5 mRNA visualized in Figure 3E and normalized to the siNS HIV-1 + condition. Error bars represent the standard deviation from three independent experiments with cells from three different donors each (One-way ANOVA; ns: not significant, * $p \leq 0.05$ and p ** $p \leq 0.01$).
